# Fluid-Screen as a real time dielectrophoretic method for universal microbial capture

**DOI:** 10.1038/s41598-021-01600-z

**Published:** 2021-11-15

**Authors:** Robert Emanuel Weber, Janusz Jurand Petkowski, Brandye Michaels, Kamil Wisniewski, Anna Piela, Slawomir Antoszczyk, Monika Urszula Weber

**Affiliations:** 1 Fluid-Screen, Inc. 100 Cummings Center, Suite 243-C, Beverly, MA 01915 USA; 2 Department of Earth, Atmospheric, and Planetary Sciences, Massachusetts Institute of Technology, 77 Mass. Ave., Cambridge, MA 02139 USA; 3 Pfizer, 1 Burtt Rd, Andover, MA 01810 USA; 4 Hener, Wrocław Technology Park, BETA Building, Room 104, Klecińska 125, 54-413 Wrocław, Poland

**Keywords:** Applied microbiology, Lab-on-a-chip

## Abstract

Bacterial culture methods, e.g. Plate Counting Method (PCM), are a gold standard in the assessment of microbial contamination in multitude of human industries. They are however slow, labor intensive, and prone to manual errors. Dielectrophoresis (DEP) has shown great promise for particle separation for decades; however, it has not yet been widely applied in routine laboratory setting. This paper provides an overview of a new DEP microbial capture and separation method called Fluid-Screen (FS), that achieves very fast, efficient, reliable and repeatable capture and separation of microbial cells. Method verification experiments demonstrated that the FS system captured 100% of bacteria in test samples, a capture efficiency much higher than previously reported for similar technology. Data generated supports the superiority of the FS method as compared to the established Plate Counting Method (PCM), that is routinely used to detect bacterial contamination in healthcare, pharmacological and food industries. We demonstrate that the FS method is universal and can capture and separate different species of bacteria and fungi to viruses, from various sample matrices (i.e. human red blood cells, mammalian cells).

## Introduction

Microbial (i.e. bacterial, viral and fungal) contamination is a serious and global threat to human health and economic development. The gold-standard method to assess the degree of microbial contamination is Plate Counting Method (PCM)^1^. Culture methods are still standard, routine techniques used in medical, pharmacological and food industries to identify bacterial contamination^2^. Unfortunately, the time-to-results for PCM is slow (days), requires the growth of the microorganisms under specific conditions used in the procedure (and may lead to the underestimation of the microorganism’s population), labor intensive, and prone to human error during media preparation, serial dilutions for sample preparation, or changing procedure conditions. Moreover, the PCM can only count and detect metabolically active cells that are capable of cell division. There is a need for new technologies that allow for faster microbial detection and assessment of microbial contamination.

Dielectrophoresis (DEP) has shown great promise for particle separation for decades (see excellent reviews^3–5^). Several DEP systems show promising potential applications in medical sciences, including drug delivery or cancer diagnostics^6–11^. However, DEP has not yet been widely applied in clinical settings. Only small sample volumes with high bacterial concentrations on the order of 10^3^–10^7^ cfu/mL have been processed, which is a limitation of the applicability of DEP microbial capture methods^12–14^. DEP separation of small cells and viral particles (~ 1 µm, and sub-µm in diameter—the size of many pathogenic bacteria and viruses) shows promise despite the fact that small bacterial particles will undergo significant Brownian motion that adds time dependent variation in their position. Thus the specificity of separation will decrease for small cells, limiting the applicability of the method (see e.g.^15^). In recent years the development of DEP separation techniques for proteins and other macromolecules also gained momentum^16–18^.

In this paper we show the new DEP bacterial capture and separation method, that overcomes those notorious limitations. We call our DEP microbial capture method Fluid-Screen (FS). We confirmed high reproducibility of the method by measuring the efficiency of the bacterial capture with the Fluid-Screen system and show the superiority of the FS method as compared to the established PCM. (“The efficiency of bacterial capture with fluid screen system” section). We establish that the FS method is universal and captures very diverse particles from different cells to viruses (“The repeatability of bacterial capture with the fluid screen system” section), and can separate bacteria from physiologically relevant fluids (i.e. human red blood cells) (“Fluid screen system captures diverse microorganisms” section). We summarize and discuss our results and the applications of the Fluid-Screen system in “Discussion” section.

## Results

In this section we describe the DEP microbial capture and separation method called Fluid-Screen (FS). First, we provide a brief overview of the design of the FS system followed a detailed verification of the FS method (“Verification of the fluid-screen method” section).

**Figure 1:**
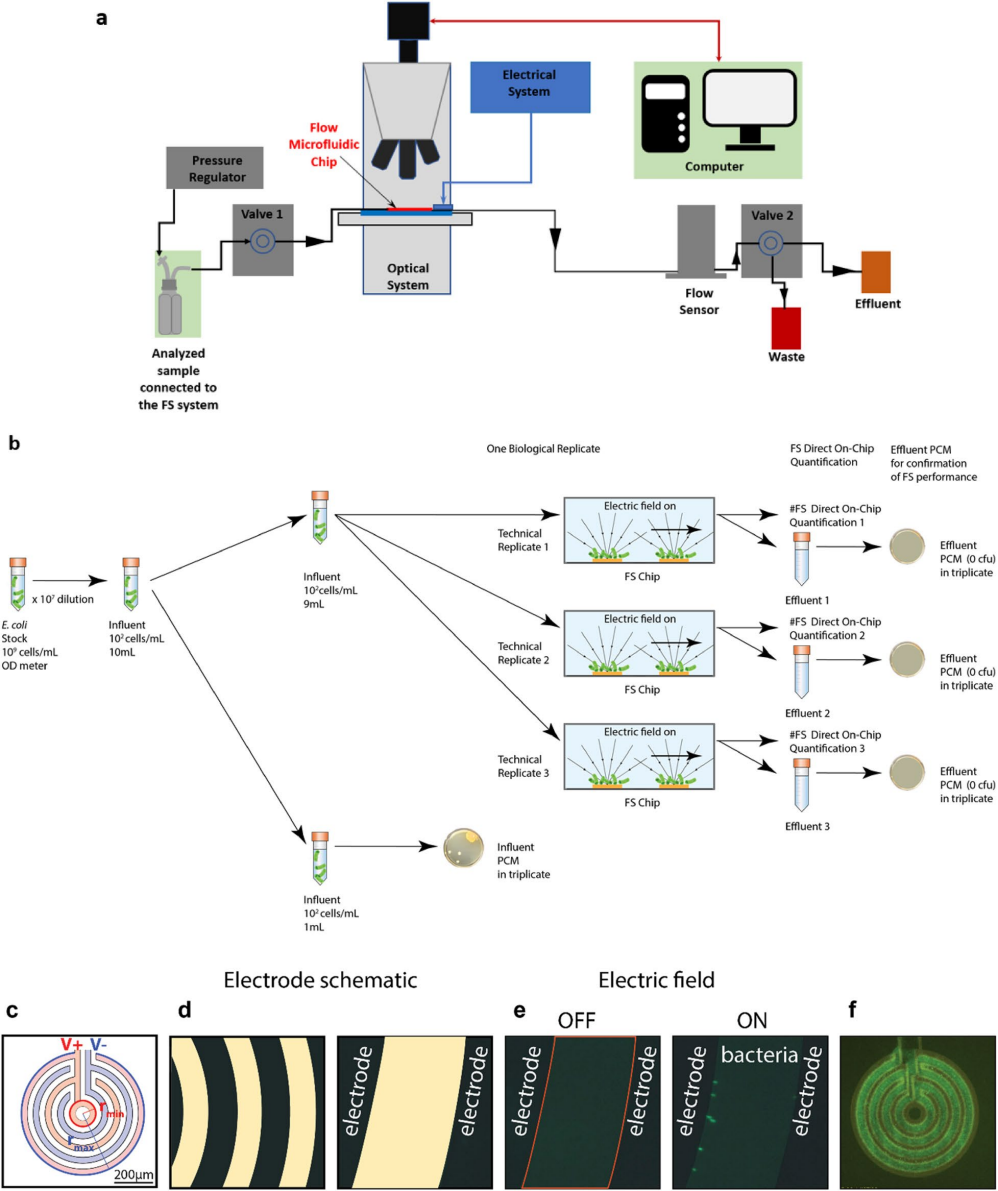
Graphical representation of the Fluid-Screen System (FS) with a schematic of the Fluid-Screen electrode design (**a**) The schematic of the FS microbial capture and separation system. The influent sample enters the chip. When the electric field is turned on, bacteria are captured on the electrodes. After the electric field is turned off, the effluent sample is collected and cultured. Sample processing of 1 mL through FS system takes approx. 4 min. (**b**) An overall schematic of the experimental procedure of bacterial capture with FS system (**c**) An overview schematic of the electrode design (system of concentric rings), with marked voltage polarity. Microbial capture experiments presented in this paper were enabled by PDMS or commercial chip fabrication (see Online Methods). (**d**) *Left panel:* Schematic of a part of an electrode system with applied alternating voltage polarity for the ring structure. *Right panel:* Zoomed-in schematic of the part of the electrode. For visual clarity the electrode is shown black, and glass, which is between electrodes, is marked yellow. (**e**) *Left panel:* Fluorescent imaging shows the electrode as black, while the glass appears light green due to autofluorescence. *Right panel: E. coli* bacteria, expressing Green Fluorescent Protein (GFP), (green dots) captured at 10 MHz and 10 Vpp on the electrode edges of the ring structure. *E. coli* bacteria align along the electrode edges when an electric field is turned on. (**f**) An overview of the electrode with *E. coli* bacteria, expressing GFP, captured from testing buffer solution spiked with bacteria. The device does not show saturation.

The overall schematic of the operation of the Fluid-Screen System (FS) is shown in Fig. 1. For detailed description of the design, geometry and operation of the Fluid-Screen electrodes, including the electric field and electric field gradients produced by the Fluid-Screen electrodes and dielectrophoretic forces involved, please see separate publications^19–21^. In brief, concentration of bacteria in a sample (influent sample containing microbes) is determined by measuring optical density (OD) and by culture and enumeration using PCM to confirm concentration in cfu/mL. The FS system pumps the influent sample through the microfluidic chip with a system of electrodes. The electrodes in the chip generate an electric field. As bacteria enter the chip, the electric field captures bacteria on the electrodes. The effluent sample is collected in a tube at the outlet of the chip.

After the Fluid-Screen System processes the entire influent sample and quantifies the number of captured bacteria, an effluent sample is collected, cultured, and enumerated using PCM for the confirmation of FS performance.

For details on the engineering design and operation see methods section below, microfabrication (Sect. S1.1) as well as microbial sample preparation (Sect. S1.2, Sect. S1.3, Sect. S1.4) and FS bacterial capture procedure (Sect. S1.5) see Supplementary Information (SI).

### Verification of the fluid-screen method

In this section we empirically verify the repeatability of the Fluid-Screen dielectrophoretic capture method (FS). First, we experimentally determine the efficiency of bacterial capture and demonstrate the superiority of the Fluid Screen capture method over the clinically established standard Plate Count Method (PCM) (“The efficiency of bacterial capture with fluid screen system” section). Secondly, we show that the FS method is equally applicable in capturing very diverse microorganisms, not only bacteria (“The repeatability of bacterial capture with the fluid screen system” section). Lastly, we verify the FS capture method, in a physiologically relevant setting by selective capture and separation of bacterial cells from human red blood cells (“Separation of bacterial cells from red blood cells” section).

#### The efficiency of bacterial capture with fluid screen system

We demonstrate that the FS system with the Fluid-Screen chip captures 100% of bacteria. Following general guidelines accepted number of colonies for reliable quantification of contamination is between 30 and 250^22^. For verification of new methods, the US Pharmacopeia (USP) requires that results are within ± 0.5 log. For this reason, the metrics of ± 0.5 log range is the basis for results evaluation with the FS method.

The achieved 100% capture efficiency is much higher than previously reported capture efficiency, for similar technology^23^. Recall that capture efficiency is dependent primarily on the electrode geometry and the electrode design^19–21^ and not on the number of bacterial cells at the input. We provide control experiments on the efficiency of bacterial capture with FS, including the estimation of lost bacterial cells during the bacterial cell capture and separation with Fluid-Screen, i.e. estimation of bacterial cell loss involving consumables and the FS system itself. We conclude that bacteria used in our experiments are not caught up in tubing and are not lost in the FS system during processing (see SI, Sect. S2.3, Fig. S5 and Table S4).

We verified the 100% capture efficiency for *E. coli-*8739 by two different counting methods.

**Figure 2:**
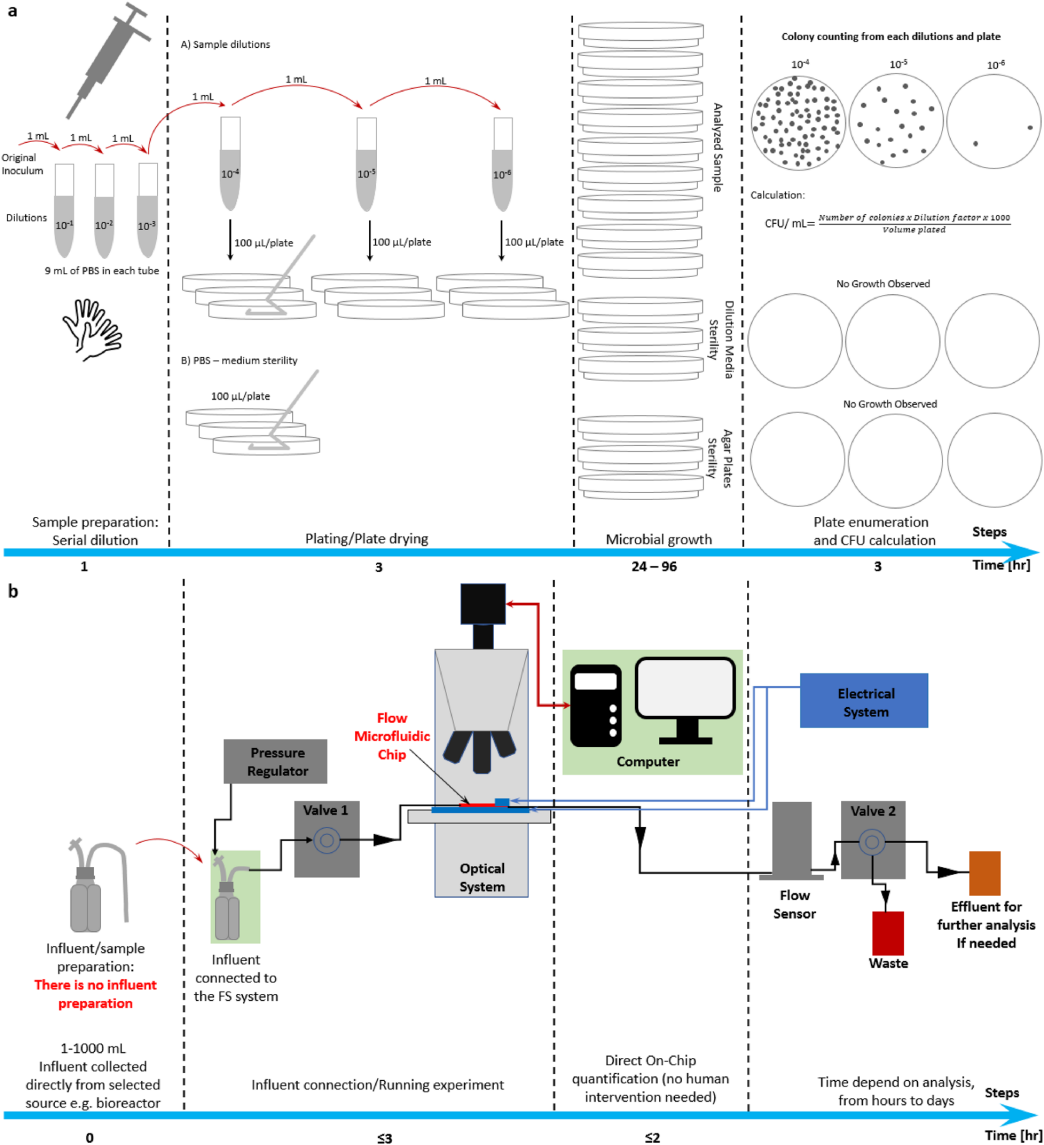
Comparison of the two procedures (**a**) clinically established standard Plate Count Method (PCM) and (**b**) FS direct “on chip quantification” approach of the Fluid-Screen microbial capture method. Schematic of an experimental setup, a part of the FS system with an indicated input (Influent), output (Effluent) sample and the Fluid-Screen Chip are shown. The FS direct “on chip quantification” is much faster, more efficient, and more reliable than the currently employed standard Plate Count Method (PCM).

First, we confirmed the capture efficiency of the unstained bacteria with the standard Plate Counting Method (PCM), we call it “PCM quantification” (Fig. 2a). The capture efficiency is defined accordingly to the following formula:

$$Capture\;efficiency = \left( {1 - \frac{{conc_{eff} }}{{conc_{inf} }}} \right) \cdot 100\%$$

conc_inf_, concentration of bacteria in the influent sample calculated based on Plate Counting Method (PCM) [cfu/mL]. conc_eff_, concentration of bacteria in the effluent sample calculated based on Plate Counting Method (PCM) [cfu/mL].

Second, we visualized captured bacteria in the sample by fluorescence microscopy with SYBR-Green staining and determined their exact number of by a direct “on chip quantification” (Fig. 2b). This process demonstrates the real number of bacteria present in the sample.

Figure 2 shows the general schematic of the FS experimental setup for both verification approaches (“PCM quantification” and “on chip quantification”). For the experiment, the influent containing *E. coli* bacteria was processed on the FS setup. In each experiment 1 mL of the effluent (output sample) was collected and plated immediately on MAC agar plates for enumeration using PCM to calculate the number of Colony Forming Units (cfus). The Electric Field settings allowing for efficient bacteria capture were determined based on a standard in-house calibration protocol (see SI, Sects. S1.2 and S1.3).

**Figure 3:**
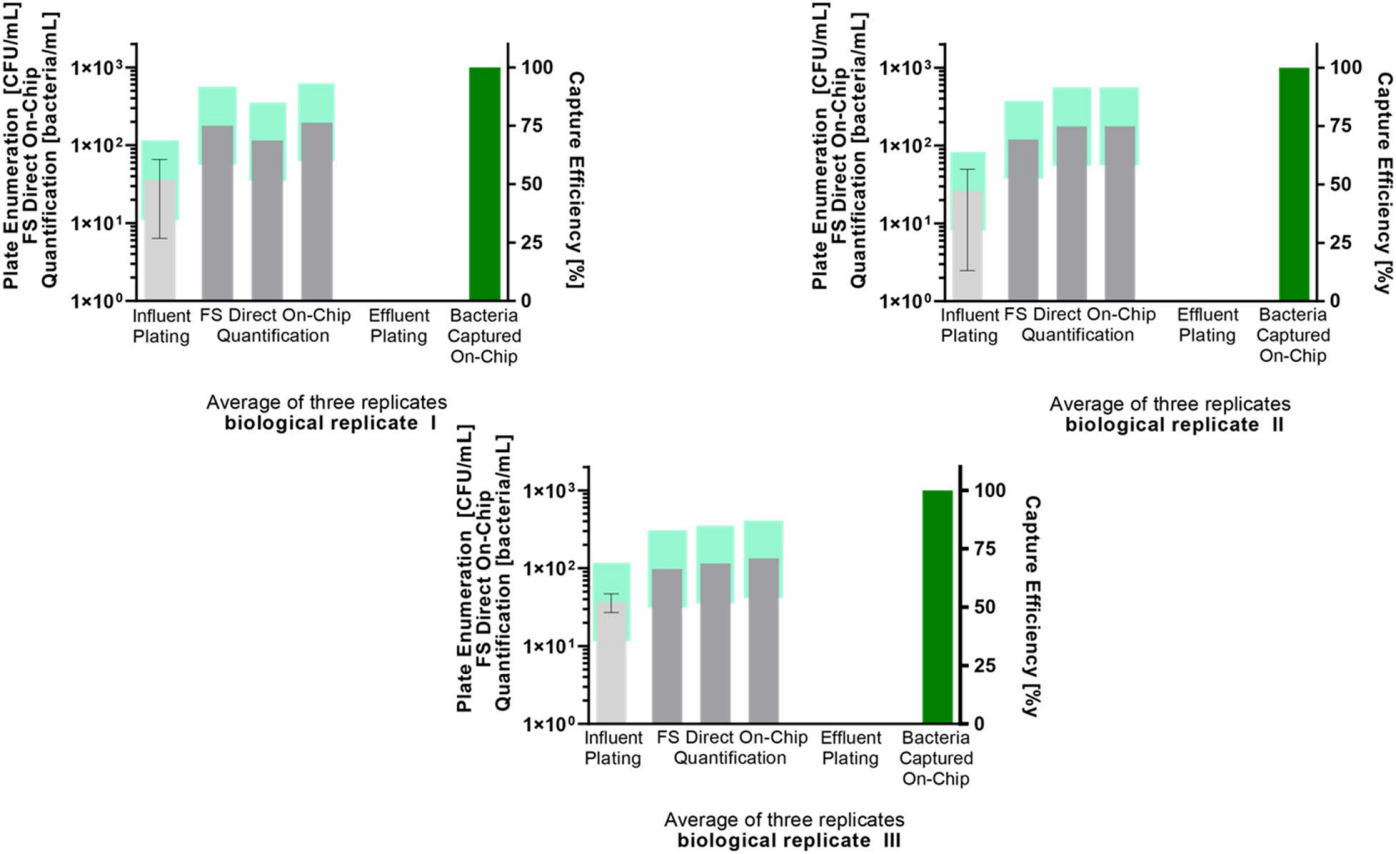
Verification of the efficiency of Fluid-Screen bacterial capture with the direct “on chip quantification” approach by standard plating method (PCM). Y axis: The number of cells in cfu mL^−1^ for plate method and cells mL^−1^ as counted by FS system. Data is presented as an average from three technical replicates per each biological replicate. The higher error bars on the plating experiments are the result of variability due to manual human intervention; the Fluid-Screen system is less prone to manual human error than PCM. Note that the FS direct “on chip” microbial count is generally higher than PCM. The higher FS count is because PCM misses cells (even if they are culturable) that are alive but, for any reason, incapable of cell division. Light green bars represent ± 0.5 log accepted by USP. The flow rate used in the Fluid-Screen bacterial capture and separation experiments is 300 µL/min.

FS demonstrates an overall 100% bacterial capture efficiency, as verified by the “PCM quantification” approach. The unstained *E. coli* capture experiment was repeated in three biological replicates (a biological replicate is new separately grown bacterial sample) with three technical replicates (a technical replicate is a triplicate repetition of the FS capture experiment, done sequentially, from the same biological replicate) per each biological replicate for a total of nine tests (Fig. 3 and Fig. S2). All three biological repeats on the FS system, in each of the nine total conducted experiments, exhibited 100% bacteria capture efficiency and repeatability (Fig. 3 and Fig. S2). Detailed data of the “PCM quantification” experiments is summarized in Table S1, including the number of bacterial colonies in the negative control, bacterial concentration in influent, bacterial concentration in effluent, and the calculated capture efficiency. The number of cfus in each influent was between 20 cfu/mL and 420 cfu/mL.

Note that in the “PCM quantification” experiment bacteria in the influent were not stained with any fluorescent stain. Lack of bacterial staining in a “PCM quantification” experiment avoids any potential growth inhibition by the fluorescent dye on MAC agar plates. For all conducted experiments, acceptable growth and variability range ± 0.5 log were reported as recommended by the USP for new method verification. The details on bacterial sample preparation are described in Online Methods, Sect. S1.2.1.

The direct “on chip quantification” of SYBR-Green stained *E. coli* is a second approach to experimentally demonstrate the FS 100% bacteria capture efficiency (Fig. 3). The direct “on chip quantification” approach is also designed to experimentally demonstrate the superiority of the FS method over “PCM quantification”. The direct FS-counted number of bacteria was determined by background subtraction from total count on the chip (Table 1).

**Table 1:** Summarized results of PCM and the direct “on chip quantification” demonstrating the FS 100% bacteria capture efficiency.

	Biol Rep 1			Biol Rep 2			Biol Rep 3		
	Tech Rep 1 [Ave cfu/mL]	Tech Rep 2 [Ave cfu/mL]	Tech Rep 3 [Ave cfu/mL]	Tech Rep 1 [Ave cfu/mL]	Tech Rep 2 [Ave cfu/mL]	Tech Rep 3 [Ave cfu/mL]	Tech Rep 1 [Ave cfu/mL]	Tech Rep 2 [Ave cfu/mL]	Tech Rep 3 [Ave cfu/mL]
**Plate-count method [cfu/mL] “PCM quantification”**									
Neg_CTRL_ (FS control buffer)	0	0	0	0	0	0	0	0	0
Influent ≤ 250 cfu/mL	70	20	17	52	12	42	65	53	20
Effluent	0	0	0	0	0	0	0	0	0
**Cap**_**eff**_** [%]**	**100**	**100**	**100**	**100**	**100**	**100**	**100**	**100**	**100**
**Direct FS baseline of particles “on-chip quantification” [particles/mL]**									
FS baseline of particles	6	15	3	4	3	12	26	13	5
**Direct total particle capture “on-chip quantification” [particles/mL]**									
Total particle capture	184	130	198	124	179	189	124	128	139
**Direct “on-chip quantification” (Total particle capture on-chip minus FS baseline of particles on-chip) [bacteria/mL]**									
Total capture	178	115	195	120	176	177	98	115	134

Cap_eff_, Capture efficiency. The flow rate used in the Fluid-Screen bacterial capture and separation experiments is 300 µL/min.

As shown on Fig. 3 Fluid-Screen direct “on chip quantification” of captured bacteria is more reliable than the standard, indirect “PCM quantification”, that requires converting the real number of captured bacteria to cfu/mL values. Most importantly, the Fluid-Screen direct “on chip quantification” yields a very small bacteria counting error. The small error is a result of a manual operation of the FS system and can be further decreased in future fully automated versions. “PCM quantification” is generally much less reliable, as it is not only indirect, but it introduces multiple sources for human error (e.g. during sample preparation and dilution, plating on agar, etc.). Therefore, standard “PCM quantification” is subjected to a large statistical error that goes beyond the ± 0.5 log range accepted by USP.

#### The repeatability of bacterial capture with the fluid screen system

We have also assessed the repeatability of the FS capture experiments. The repeatability verification result shows that the FS system demonstrates very high repeatability in the capture and quantification of bacteria. Moreover, the method is more accurate than the required ± 0.5 log accepted by the USP for new method verification (see Fig. 3 and SI Sect. S2.2; Fig. S3, Fig. S4, Table S2).

In conclusion, the FS system has demonstrated critical functionality in capturing all bacteria that were present in the test samples, at various bacterial concentration ranges. The 100% bacterial capture efficiency was verified both by a standard PCM and by high-performance direct on-chip quantification. As presented for *E. coli,* the FS system demonstrates very high repeatability of bacterial capture. In addition, it allows to quantify the directly FS-counted number of microorganisms in analyzed samples.

#### Fluid screen system captures diverse microorganisms

Fluid-Screen technology can capture and detect very diverse microorganisms. It is not limited to *E. coli.* It captures both Gram-negative and Gram-positive bacteria, multiple bacterial morphologies, and both individual bacteria and cell aggregates, including bacteria that cannot be cultured or do not culture easily (e.g. certain strains of *Mycoplasma hyorhinis* and *Legionella pneumophila*). Herein we demonstrate that not only Gram-negative, Gram-positive, bacilli, and cocci bacteria, but also yeast and mold (including conidia, conidiophores and hyphae), as well as viruses respond to the electric field, and therefore can be efficiently captured and separated. A total of 40 different species of microorganisms were tested providing the proof of concept for the broad applicability of the FS system. All microorganisms responded to the electric field and were captured, as verified by optical microscopy (Table 2, Table S3). The detailed statistical analysis of the capture efficiency of the other microbial species is going to be presented in future dedicated follow up studies.

**Table 2:** Microorganisms captured using FS universal microbial capture approach.

Capturing of microorganisms from a variety of taxa				
Tested organism	Taxonomy domain of life	Microorganism differentiation	Media	Medical, industrial or environmental significance of the tested organisms (after https://www.atcc.org)
*E. coli*-8739	Bacteria	Gram (−)	PBS Drug substance (concentrated protein solution) Mammalian cell culture medium	Tests for microbial contamination
*P. aeruginosa-*9027	Bacteria	Gram (−)	PBS Drug substance (concentrated protein solution) Mammalian cell culture medium	Tests for microbial contamination
*B. cereus* 13061	Bacteria	Gram (+)	PBS	(see Table S3)
*B. coagulans* BAA-738	Bacteria	Gram (+)	PBS	Potential probiotic
*B. circulans* 9500	Bacteria	Gram (+)	PBS	Various environmental applications including biodegradation, drain cleaning and degreasing, septic tank maintenance, as well as waste and wastewater treatment; Pharmaceutical product contamination
*B. megaterium* 14581	Bacteria	Gram (+)	PBS	Used as an alternative for high yield intra- and extracellular protein synthesis and in quality control
*B. oleronius* 700005	Bacteria	Gram (+)	PBS	Establishment of sterilization conditions
*B. subtilis* 6051 (including endospores)	Bacteria	Gram (+)	PBS	Pharmaceutical product contamination
*A. brasiliensis*-16404	Eukaryota Multicellular (Fungus)	Mold	PBS Drug substance (concentrated protein solution) Mammalian cell culture medium	Tests for microbial contamination
*C. albicans*-10231	Eukaryota single-celled (Fungus)	Yeast	PBS Drug substance (concentrated protein solution) Mammalian cell culture medium	Tests for microbial contamination
Human adenovirus 5 VR-5^24^	N/A	N/A	PBS Mammalian cells cultured medium	Virucide testing Respiratory research

All the organisms listed in this table and Table S3 in the SI responded to the electric field and were captured on the FS electrode. See Table S3 in the SI for a complete list of microorganisms captured using FS universal microbial capture approach.

#### Separation of bacterial cells from red blood cells

Fluid-Screen system is capable of not only universal capture of diverse microbial organisms, it can also separate them, and selectively capture only microbial species of interest. In this section we illustrate the selective capture capability of FS system in a physiologically relevant setting, by capturing *E. coli* bacteria from human red blood cells.

A number of early studies from the late 90 s and early 2000s have demonstrated DEP separation of eukaryotic cells^25–27^, including DEP separation of bacteria from blood cells^28^, cancer cells from blood cells^29,30^ and cancer cells from CD 34^+^ hematopoietic stem cells^31,32^.

Separation of bacteria from blood is challenging because blood is a complex fluid. Every microliter of blood contains about 5 million red blood cells, in addition to platelets, white blood cells, and proteins. Blood plasma is a high ionic solution containing proteins and ions, which can add to electric screening or chemical non-specific binding, which in turn could lower the efficiency of FS DEP capture. Overcoming such challenges and achieving reliable and efficient detection of bacteria is crucial in clinical diagnostics. For example, to diagnose sepsis, it is required to detect a single bacterium from 1 mL of blood. Efficient and accurate separation and capture of bacteria in blood can result in automated and fast sample preparation on chip.

**Figure 4:**
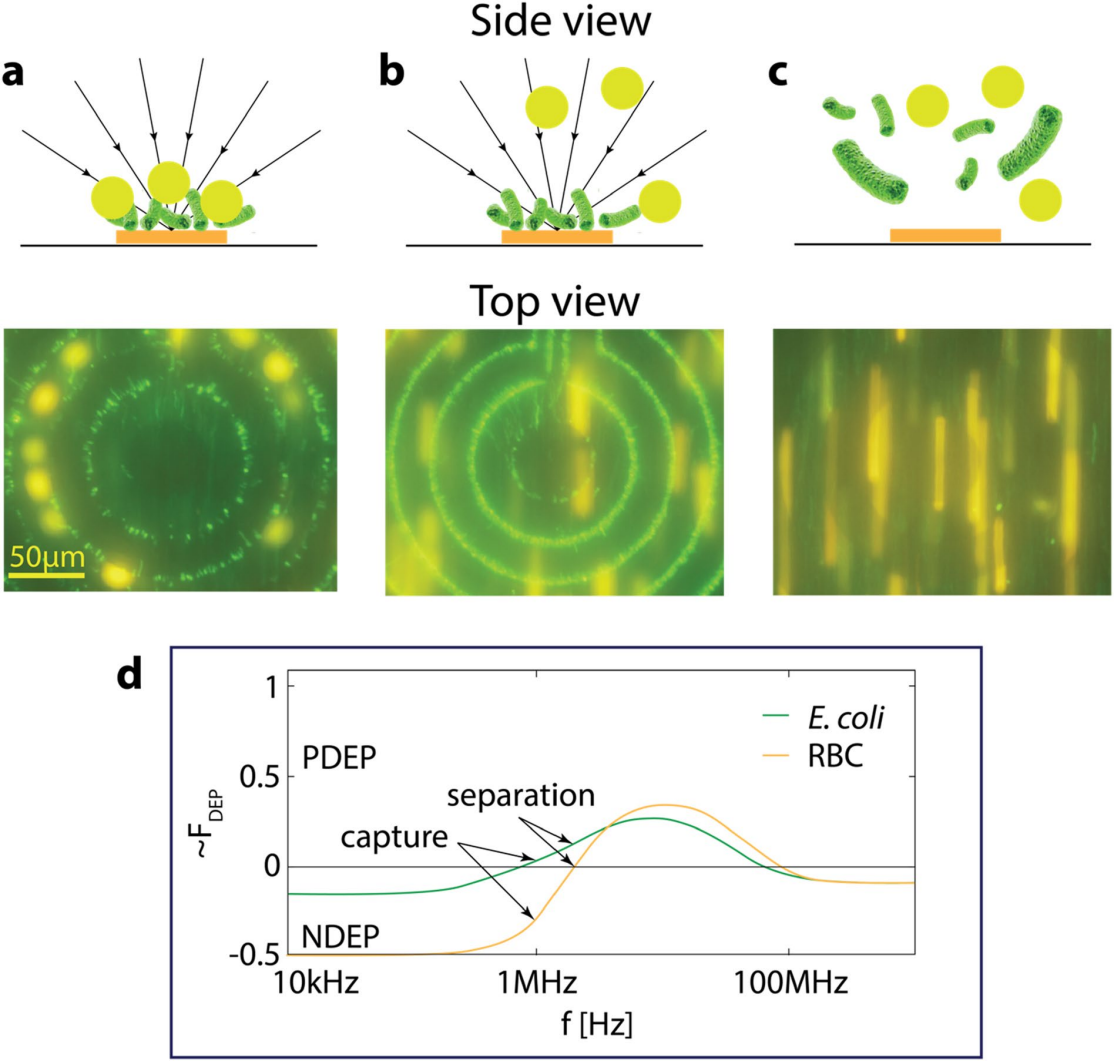
Capture and release of *E. coli* and RBCs from 10 times diluted human blood, (**a**) simultaneous *E. coli* (green) and RBC (yellow) dielectrophoresis capture at *f*_2_ = 2 MHz with FS chip (orange bar) (**b**) simultaneous *E. coli* (green) dielectrophoresis capture at *f*_1_ = 10 MHz and RBC (yellow) flow separation, (**c**) *E. coli* (green) and RBC (yellow) flow in the absence of electric field. (**d**) Calculated DEP force (PDEP) for *E. coli* and RBCs. Experimental results confirm the theoretical predictions. In solution conductivity 100 mS m^−1^ *E. coli* bacteria have a positive DEP force at 2 MHz and at 10 MHz, calculated DEP force for RBCs, calculations show DEP force = 0 at 2 MHz and a positive DEP force at 10 MHz. The DEP force was calculated using model^33^. The flow rate used in the FS red blood cell capture and separation experiments is 0.02 µL/min. This slow flow was chosen on purpose to optically verify separation of *E. coli* bacteria from red blood cells. Further work is needed to optimize the assay to achieve the standard FS flow of 300 µL/min.

We have separated *E. coli* bacteria from human red blood cells. The capture and separation of *E. coli* from red blood cells was performed using PDMS FS chips (see Online Methods). The results of the *E. coli* capture from diluted serum in the presence of red blood cells (RBCs), together with the dielectrophoretic conditions of the bacterial capture are summarized on Fig. 4. Our results show *E. coli* capture and separation from a human red blood cells sample. The detailed statistical analysis of the capture efficiency and separation of bacterial cells from physiologically relevant fluids is a domain of future dedicated work.

In conclusion, we have verified the approach and shown that the Fluid-Screen dielectrophoretic method for universal microbial capture is characterized by high efficiency of capture, with no false negatives or false positives. We showed that the method is reliable, with high repeatability and fast response and operation. We showed that FS can work on diluted physiological solutions (i.e. human blood) and showed high yield of separation in the presence of blood cells, thus meeting the high selectivity requirements. Most importantly from the clinical perspective, it can process high volumes of liquid to meet clinical testing standards. While the FS is not yet ready to be used in the clinical setting, the technology has clear potential future clinical applications. Further testing on the whole blood samples, followed by the clinical trial campaign, should be performed to fully demonstrate the method’s application in clinical setting.

## Discussion

We have presented Fluid-Screen (FS)—a new dielectrophoretic method for universal microbial capture. We have extensively verified Fluid-Screen performance in terms of efficiency and repeatability of *E. coli* capture. The FS method shows fast response and operation (processing of 1 mL of sample through FS system takes approx. 4 min) and is highly reliable.

The FS method captures 100% of the bacteria present in all tested samples (Table 1; Table S1), as verified both by the established PCM and by direct bacteria on-chip-quantification (Fig. 2 and Fig. 3). Fluid-Screen demonstrates high repeatability of bacterial capture process that provides high levels of confidence (Fig. S3), as exemplified by the fact that the Fluid-Screen method meets the error range of ± 0.5 log recommended by the USP for new method validation.

Here we demonstrated the superiority of the Fluid-Screen microbial capture over the standard culture method (i.e., PCM). The PCM is a multistep, indirect method of assessing the degree of bacterial contamination. The Fluid-Screen method gives a direct number of cells in the sample without the need for a plating step. Moreover, the established PCM is only capable of detecting and counting live bacteria that form colonies on the plate. FS on the other hand captures and detects all cells, including alive colony forming ones, alive but metabolically inactive, spores etc., all of which nevertheless can cause serious health hazard if left undetected. The ability of reliably capturing and detecting cells in all their various metabolic states is a serious improvement over the currently used PCM (Fig. 3).

We have demonstrated that the FS method can capture a plethora of microorganisms from variety of taxa (Table 2 and Table S3), confirming that the FS method is a universal microbial capture approach suitable for capture and identification of any microorganism, from bacteria to single-cell and multi-cellular eukaryotes, to even viruses (Table 2 and Table S3). We note that some of the captured microorganisms are difficult to identify and count by other methods. FS technology does not require microorganisms to be able to grow in the laboratory conditions, and as such FS can capture and identify microorganisms for which the classical PCM technique would never work.

We have also demonstrated that FS can capture and separate *E. coli* bacteria from human red blood cells (Fig. 4).

The versatility of the Fluid-Screen microbial capture method makes it potentially applicable in a variety of clinically relevant settings, including in healthcare, food, and pharmacology industries as well as in environmental studies, anywhere where fast and reliable detection of a broad range of microbial contaminants is paramount.

Taken together, FS technology is not only devoid of the shortcomings of the PCM, but it is also faster and much more reliable (due to reduction of human intervention and human errors) in the estimation of the bacterial contamination. FS can demonstrate a clear advantage over plate count enumeration methods, with higher levels of accuracy indicated by a true, direct bacterial count from the tested samples, and a reduced variability between samples.

The FS method is semi-automatic. It does not require intensive labor (in contrary to PCM) or training and has much higher reproducibility.

The engineering design of the Fluid-Screen System is very versatile, allowing for ease of modification and future improvements, including, but not limited to, automation of the entire process (to further reduce operational errors), miniaturization for environmental studies, on-line (continuous monitoring) or in-line testing etc.

## Materials and methods

### Engineering design and operation of fluid-screen system

**Figure 5:**
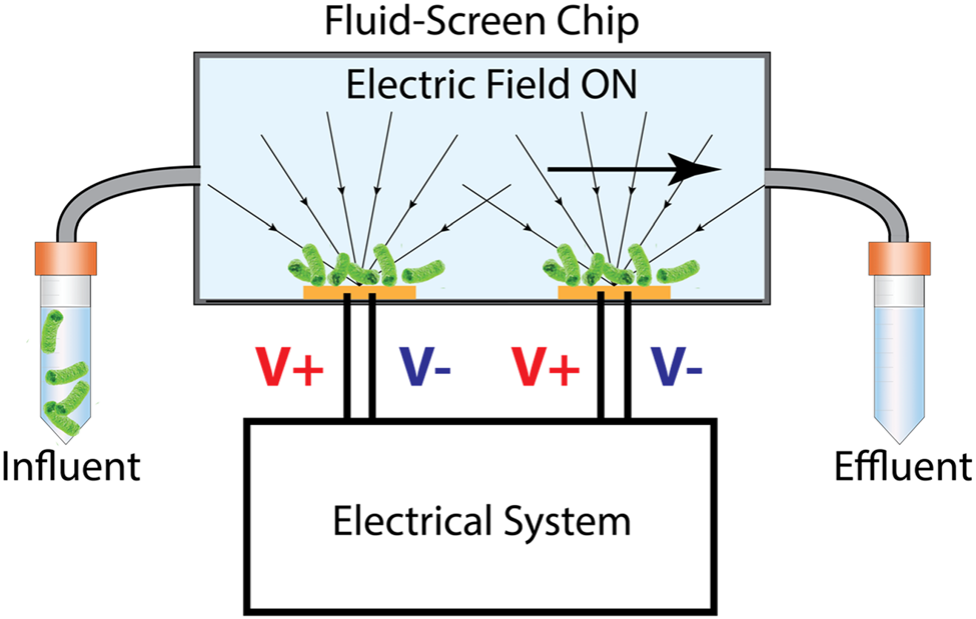
Schematic of the Fluid Screen chip in operation: microchannel, electrodes on the bottom (yellow), influent and effluent samples are connected via tubing. The Electrical System applies alternating voltage V+, V− to the electrodes generating electric field inside the microfluidic channel. The main arrow shows the direction of fluid flow through the chip from inlet (left) to the outlet (right). Arrows pointing to the electrodes show the directions of the dielectrophoretic force while the electric field is turned on.

Figure 5 illustrates a schematic diagram of the Fluid-Screen chip for capturing bacteria from a sample using system in Fig. 1. As shown, in the presence of an electric field generated using electrode (orange bar), bacteria (green) are attracted to the electrode by a positive DEP force (arrows) acting on the bacteria in the sample. Sample components are introduced to the Fluid-Screen chip from an influent sample. The sample flows past the electrode in the microfluidic chip at a predetermined flow rate. Sample components not captured by the electrode flow to the effluent sample.

**Figure 6:**
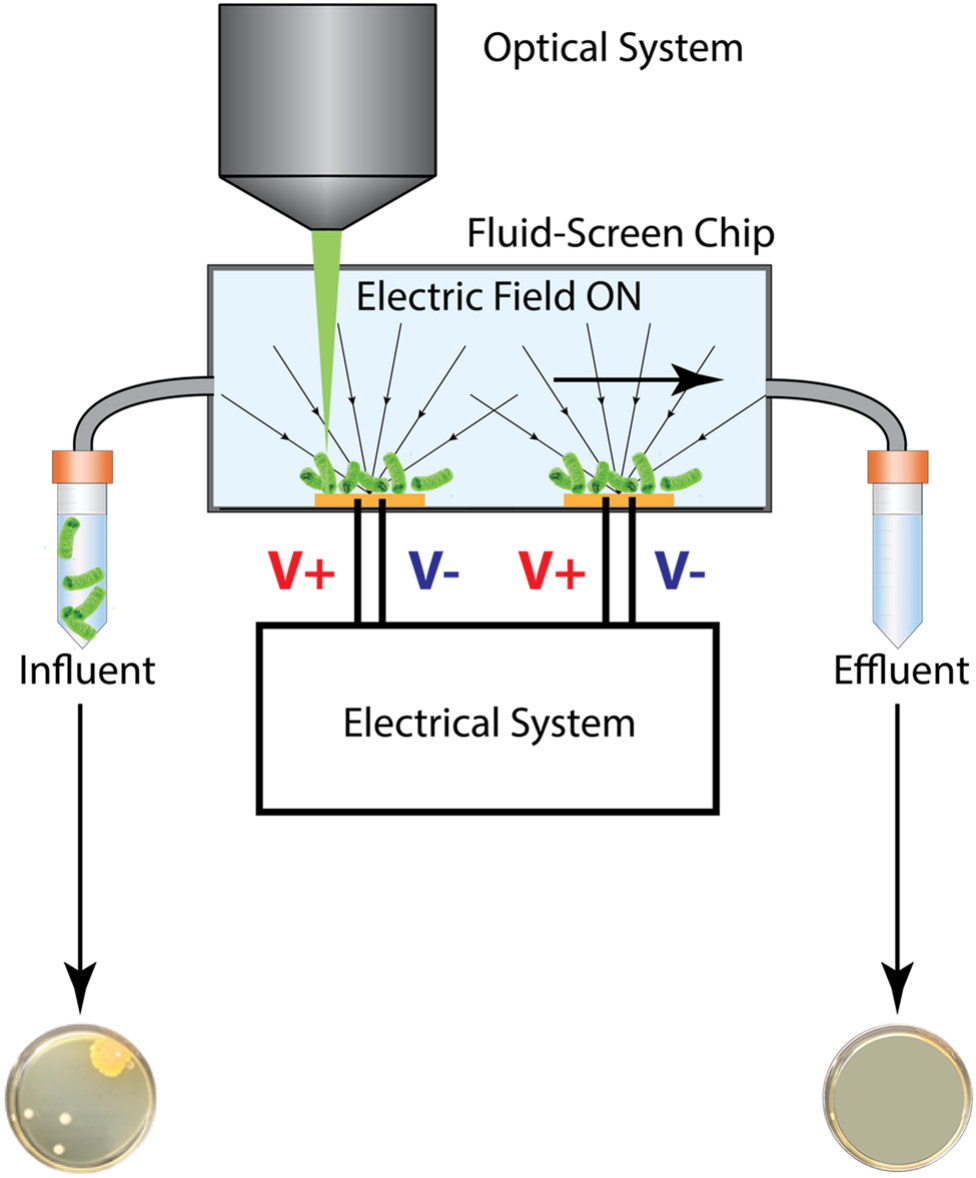
Schematic of the Fluid Screen experimental setup: microchannel, electrode on the bottom with +V and −V contacts, electrodes are connected to a function generator and an oscilloscope, bacterial motion is observed with a microscope and registered with a camera. Arrows pointing to the electrodes show the directions of the dielectrophoretic force while the electric field is turned on.

Figure 6 illustrates a schematic diagram of capturing bacteria from a sample using system in Fig. 1. The bacteria captured on the electrodes are imaged using the optical system to perform a direct on-chip quantification. Influent sample and effluent sample are plated on agar plates for PCM.

The Fluid Screen electrode system is the ring structure that consists of concentric rings (Fig. 1). Every second ring is connected to the same potential. The outer radius of the inner ring is 50 µm and the outer radius of the outer ring is 250 µm. The ring structure captured 100% of bacteria according to PCM (Table 1; Table S1) which is much higher than previously reported capture efficiency results for similar technology^23^. To achieve 100% capture efficiency (as verified by PCM) a proper configuration of the FS system and the FS chip is required. If those are not configured properly then the capture efficiency of the FS system is severely diminished.

The experimental setup (Fig. 1) consists of the fabricated chip with the electrode structure located on the bottom of the microfluidic channel with fluid flow above the electrode. Both contacts are connected to opposite polarities with Fluid-Screen custom made interface, SMA cables, Fluid-Screen custom-made amplifier, and function generator (Siglent SDG5162, USA). Bacterial capture is observed with a fluorescent microscope (Olympus BX63, USA).

Flow was controlled by Elvesys’ microfluidics system and kept constant throughout the entirety of the experiment. During the experiment the measurement apparatus was controlled by the LabView System Controller and the scan image was obtained using Olympus CellSens Software.

The data on microfabrication of the chip, microbial sample preparation, and microbial capture procedure are provided in the Supplementary Information (SI).

## Supplementary Information

Supplementary Information.
