# Supplementary material for: Fluid-Screen as a real time dielectrophoretic method for universal microbial capture
Source: Sci Rep. 2021 Nov 15;11:22222. doi: 10.1038/s41598-021-01600-z (PMC8594773; doi:10.1038/s41598-021-01600-z)
Supplement: Supplementary file 1 — Supplementary Information. [file 41598_2021_1600_MOESM1_ESM.docx]

Supplementary Information for

**Fluid-Screen as a Real Time Dielectrophoretic Method for Universal Microbial Capture**

Robert Emanuel Weber^1^, Janusz Jurand Petkowski^2^, Brandye Michaels^3^, Kamil Wisniewski^4^, Anna Piela^4^, Slawomir Antoszczyk^1^, Monika Urszula Weber^1^*

**Affiliations:**

^1^ Fluid-Screen, Inc. 100 Cummings Center, Suite 243-C, Beverly, MA 01915, USA
^2^ Department of Earth, Atmospheric, and Planetary Sciences, Massachusetts Institute of Technology, 77 Mass. Ave., Cambridge, MA 02139, USA
^3^ Pfizer, 1 Burtt Rd, Andover, MA 01810, USA
^4^ Hener, Wrocław Technology Park, BETA Building, Room 104, Klecińska 125,
54-413 Wrocław, Poland

* Correspondence to: [monika.weber@fluid-screen.com](mailto:monika.weber@fluid-screen.com)

# 1. Supplementary Methods:

## 1.1 Microfabrication

The procedure for fabrication of Polydimethylsiloxane (PDMS) devices is very similar to what is described in the literature^2^ regarding soft lithographic techniques.

The steps of PDMS chip fabrication and assembly are the following:

1. Preparation of PDMS mixture with 1:10 curing agent: PDMS mixed in the fume hood, placed in the desiccator for 30 minutes to remove bubbles.

2. PDMS mixture poured onto a wafer mold wrapped in aluminum foil and placed in the desiccator for 30 minutes to remove bubbles.

3. Si wafer mold with PDMS layer placed on the hotplate at 80° C for five hours.

4. Cooled PDMS peeled off the wafer mold and punched with the hole puncher.

5. Glass substrate and PDMS exposed to oxygen plasma at 350 mTorr at 200W for 30s.

6. Microfluidic device assembled and bonded for five hours at 60° C.

Figure S1 shows the process flow to fabricate circular microelectrodes using a bilayer lift-off process. Note that PDMS device is used only in experiments of separation of *E. coli* from red blood cells (as shown in section 2.1.3 of the main text). All other microbial capture experiments presented in this paper were enabled by commercial chip fabrication.


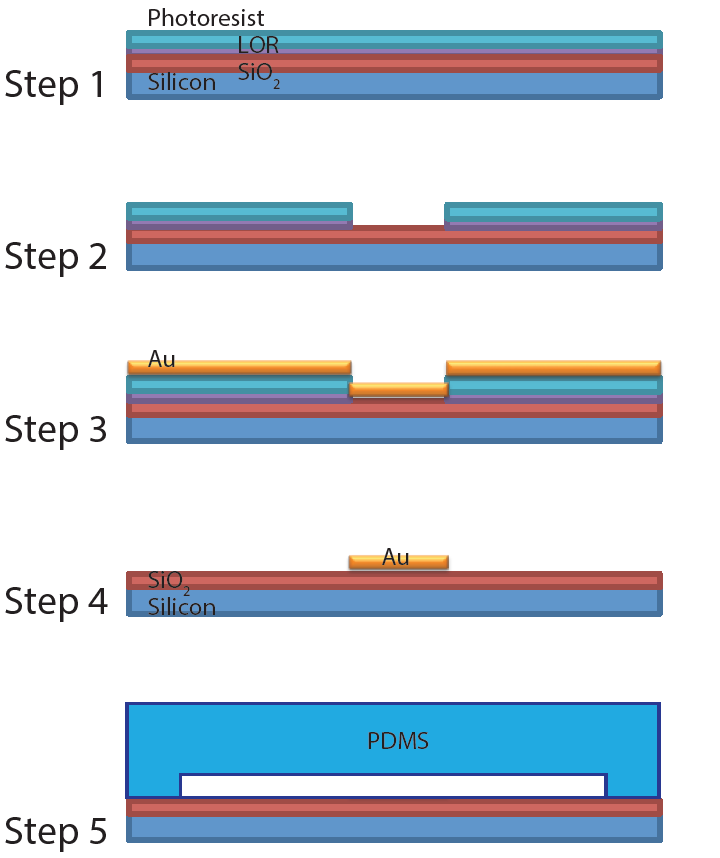


**Figure S1.** Electrode pattern fabrication using a bi-layer lift-off process, Step 1: on a silicon wafer with 3µm SiO_2_ spin *LOR*10*A* and *S*1808, Step 2: expose and develop features with *MF*312 : *DI* 1 : 1, Step 3: evaporate Ti 5 nm and Au 40 nm, Step 4: lift off gold using 1165 Microposit remover, Step 5: PDMS bonding to silicon substrate with oxygen plasma at 100 W for 30 seconds at 350 mTorr.

## 1.2 *E. coli* Bacterial Sample Preparation

### 1.2.1 Unstained *E. coli* Sample Preparation for Capture Efficiency Assessment

The *E. coli-*8739 strain was obtained from ATCC and cultured on Tryptic Soy Agar (TSA) and MacConkey (MC) agar plates at 37°C in aerobic conditions following the Harmonized USP/EP/JP.

The influent (input) sample the unstained *E. coli* capture experiment (Figure S2) was prepared in the following way. A day before the FS bacterial capture experiment on the FS system *E. coli*-8739 was re-streaked, using sterile inoculation loop, on TSA agar plates by progressive dilutions of an inoculum from a single colony on agar plates. From an overnight culture a large scoop of bacteria, avoiding taking bacteria from a biofilm area on the plates, was taken using sterile inoculation loop and suspended in 2 mL of PBS 1:1000 (Fluid Screen standard dilution testing buffer). A stock sample concentration of 10^7^-10^8^ bacteria/mL was determined using OD (optical density, CO8000 Cell Density Meter, Biowave) meter at 600 nm, and plated immediately after preparation in serial 10x dilutions (e. g. 10^-4^, 10^-5^, and 10^-6^) and in triplicates on MAC agar plates to confirm bacteria concentration in cfu/mL. The stock sample was diluted by serial 10x dilution to the final bacterial concentration up to 250 cfu/mL. All experiments were conducted at room temperature. To prepare stock and experimental samples buffer and media were warmed up to room temperature prior to use.

To track any possible changes in bacteria concentration in influent, i.e. bacterial death over time, 100 µL aliquots of influent sample were plated. Each 100 µL influent aliquot was plated without and with single 10x dilution in triplicates on MAC agar plates to confirm bacteria concentration in cfu/mL.

### 1.2.2 Sybr Green I Stained *E. coli* Sample Preparation for Capture Efficiency Assessment

The *E. coli-*8739 was obtained from ATCC and cultured on Tryptic Soy Agar (TSA) or MacConkey (MAC) agar plates at 37°C in aerobic conditions as recommended by the Harmonized USP/EP/JP.

The influent (input) sample for the stained *E. coli* capture experiment (Figure 3 in the main text) was prepared in the following way. A day before the experiment *E. coli*-8739 was re-streaked on TSA agar plates by the progressive dilution of single colonies on agar plates using a sterile inoculation loop. From an overnight culture a large scoop of bacteria (while avoiding taking bacteria from a biofilm area on the plates) was taken using a sterile inoculation loop and suspended in 2 mL of PBS 1:1000 (Fluid Screen standard dilution testing buffer, 1X PBS (Life Science) diluted 1:1000 in UltraPure DI water, herein 0.001xPBS). A stock sample concentration of 10^7^-10^8^ bacteria/mL were determined using the OD meter at 600 nm and plated immediately after preparation of serial tenfold dilutions (e.g. 10^-4^, 10^-5^, and 10^-6^) on MAC agar plates to confirm bacteria concentration in cfu/mL. The final concentration of influent up to 250 cfu/mL was achieved by serial tenfold dilution of the stock sample. Meanwhile, standard cleaning procedure of the FS system and chip was performed. To confirm that FS system is sterile before sample processing, unit was flushed with sterile DI UltraPure Water, of which 5 mL was collected and plated on agar media (TSA, MAC, MSA, SDA and CA), and 1 mL of Sybr Green I diluted 1:1000 in PBS was processed through the FS unit. The FS system was rinsed with DI UltraPure water to remove residue fluorescent dye, the influent was processed through the FS system, and 1 mL of the effluent was collected and plated immediately on MAC agar for enumeration using PCM (to calculate the number of cfu). The electric field (EF) settings were determined based on a standard in-house calibration protocol and optimized for tested bacteria. Bacteria captured on the FS system chip were stained with Sybr Green I and counted based on background subtraction. The percentage of capture efficiency was calculated based on formula presented in section 2.1.1 of the main draft. The experiment was carried out in three biological replicates with three technical replicates per each biological replicate (Figure 3 in the main text; see also Figure S2). All experiments were conducted at room temperature. To prepare stock and experimental samples, the buffer and media were warmed up to room temperature.

### 1.2.3 *E. coli* Sample Preparation for FS Repeatability Studies

The *E. coli-*8739 was obtained from ATCC and cultured on Tryptic Soy Agar (TSA) agar plates at 37°C in aerobic conditions as recommended by the Harmonized USP/EP/JP.

The *E. coli* samples for the repeatability experiments were prepared in similar fashion to the samples for the other experiments (see Section S1.2.1 and Section 1.2.2). A day before the experiment *E. coli*-8739 was re-streaked on TSA agar plates by the progressive dilution of an inoculum (from a single colony) on agar plates using a sterile inoculation loop. From an overnight culture a, large scoop of bacteria (while avoiding taking bacteria from a biofilm area on the plates) was taken using a sterile inoculation loop and then suspended in 2 mL of 0.001xPBS. A concentration of tested sample 10^5^-10^6^ bacteria/mL were determined using OD meter at 600 nm and confirmed by plating (immediately after preparation) in serial tenfold dilutions on TSA plates. To visualize the bacterial response to an electric field, 1 mL of the tested sample was stained with 1 μL of Sybr Green. Using a micropipette, 2 μL of the stained sample were loaded to each channel of the chip under static conditions. When the electric field (EF) was on, bacteria were captured, and fluorescent images of the entire spiral electrode were taken to quantify the true number of bacteria. The EF settings were determined based on a standard in-house calibration protocol and optimized for the tested bacteria. The experiment was carried out on in two biological samples with 12 technical replicates per each biological sample. Total repeatability was evaluated on eight chips, with three channels per chip. For the null control image, the whole spiral electrode from each chip (without bacteria, using the same fluorescent acquisitions) was used to analyze the samples in the FS chip channels.

All experiments were conducted at room temperature. To prepare stock and experimental samples, the buffer and media were warmed up to room temperature.

### 1.2.4 Cell Culture and Sample Preparation for Estimation of Losses of Bacterial Cells During the Bacterial Cell Capture and Separation with Fluid-Screen

*E. coli-*8739 was obtained from ATCC and cultured on TSA plates at 32°C in aerobic conditions as recommended by the Harmonized USP/EP/JP. A day before the experiment, *E. coli*-8739 was re-streaked on TSA plates by the progressive dilution of an inoculum on agar plates using a sterile inoculation loop. From an overnight culture a large scoop of bacteria (while avoiding taking bacteria from a biofilm area on the plates) was taken using a sterile inoculation loop and suspended in 2 mL of 0.001xPBS. A stock sample concentration of 10^7^-10^8^ bacteria/mL was determined using OD meter at 600 nm. The final concentration of influent (input sample which is processed on the FS Unit) was achieved by serial tenfold dilution of stock sample.

To verify agar media sterility, three plates from each batch were incubated in 37°C in aerobic conditions for five days. To verify aseptic sample preparation conditions, 10 mL/plate of sterile 0.001xPBS was filtrated through 0.45 µm Analytical Filter Units (Thermo Fisher), plated on TSA plates and incubated at 32°C in aerobic conditions for five days. In both cases “zero” growth must be observed.

### 1.2.5 Filtration Method for Influent and Effluent Plating

Following the experiment, the entire 10 mL effluent is filtrated through the 0.45 µm Analytical Filter Units, plated immediately on TSA plates and incubated at 32°C for up to three days, then enumerated using Plate-Count Method to calculate the number of cfu. Similarly, 1 mL of remaining influent sample is added to 9 mL of 0.001xPBS and plated in the same way.

## 1.3 Other Microbial Samples Preparation

All microorganisms and adenovirus sample were bought from ATCC, in freeze-dried condition. Bacteria, yeast and fungus (black mold) cultures were started and propagated at Fluid-Screen’s laboratory following supplier’s recommendation.

Growth conditions: *E. coli* (ATCC 8739), *S. aureus* (ATCC 6538), *P. aeruginosa* (ATCC 9027), *B. subtilis* (ATCC 6633), *C. albicans* (ATCC 10231) and *A. brasiliensis* (ATCC 16404) were selected from USP <61> and <62>. Bacteria, yeast and fungus grew in aerobic conditions between 20-37°C and were cultured no longer than five consecutive passages in the recommended culture conditions. All tested microorganisms were cultured on Tryptic Soy Agar (TSA) or MacConkey Agar (MAC), Mannitol Salt Agar (MSA) and Cetrimide Agar (CA) for bacteria and Sabouraud Dextraose Agar (SDA) for yeast and fungus as recommended by the Harmonized USP/EP/JP.

Samples for other microorganisms were prepared in an analogous way to the *E. coli* samples for the other experiments (see Section S1.2.1 and Section 1.2.2). A day before the experiment all microorganisms were re-streaked on appropriate agar plates, by the progressive dilution of an inoculum (from a single colony) using a sterile inoculation loop.

Analyzed samples ~10^7^ microorganisms/mL (except *A. brasiliensis*) were prepared from an overnight culture in sterile PBS 1x (Life Science) diluted 1:1000 in DI UltraPure water (Life Science). *A. brasiliensis* sample was less concentrated (~10^3^ spores/mL) and contained mix of spores (conidia), conidiophores and hyphae. Bacteria and yeast concentration were determined by OD meter at 600 nm. To evaluate that the microorganisms responded to an electric field (EF), stock samples were spun down, the supernatant discarded, then suspended in CHO cell matrix diluted 1:100 in DI UltraPure water. For optical visualization, microorganisms (except *A. brasiliensis*) were stained with Sybr Green I (working solution 1:1000) by adding the dye directly to the sample. Because Sybr Green I does not penetrate *A. brasiliensis* conidia and they are large enough, mold was observed in a bright field. Control samples were prepared in the same way- microorganisms were suspended in PBS diluted 1:1000 in UltraPure DI water and were stained with Sybr Green I. The 3 µL of sample was applied to the FS system under static conditions. A video demonstrating that tested microorganisms respond to the EF were collected in triplicate. The EF settings were determined based on a standard in-house calibration protocol and optimized for the tested microorganisms.

All experiments were conducted at room temperature. To prepare stock and experimental samples, the buffer and media were warmed up to room temperature.

For all FS experiments sterile conditions were verified and maintained. To verify agar media sterility, three plates from each batch were incubated at 37°C in aerobic conditions for five days. To verify aseptic sample preparation of test samples, 100 μL of the Fluid-Screen sample buffer used to prepare samples were plated on TSA, MAC, MSA, CA and SDA and incubated in 37°C in aerobic conditions for five days. The sterility test of the FS system, media and other materials showed no growth.

**Statistics and Calculations**

For statistical analysis we used Microsoft Excel and GraphPad Prism. The acceptable growth and viability variance range of +/- 0.5 log recommended by the USP were calculated using the following example method: A value from plate enumeration or on-chip quantification was e.g. A, so for + 0.5 log value: Ax3.2=B, and for - 0.5 log value: A/3.2=C. All +/- 0.5 log values were calculated in the same way.

## 1.4 Red Blood Cells Sample Preparation

The human blood sample (donated by the co-author MUW) was spun to separate RBCs (red blood cells).  RBCs were fluorescently stained for visual clarity. Stained RBCs were added into the sample that was diluted 10 times with deionized water. *E. coli* expressing GFP was grown on LB broth, resuspended in testing buffer and added to the blood sample.

## 1.5 Fluid-Screen Bacterial Capture Experimental Procedure

*E. coli*-8739 stock sample was prepared in ~10^7^cfu/mL in testing buffer (Fluid Screen standard dilution buffer) by measuring absorbance on OD meter at 600 nm. The final concentration of Influent, up to 250 cfu/mL was achieved by serial 10x dilution of stock sample.

The 1 mL of sample was applied to the FS system under flow conditions and electric field ON (voltage and frequency settings optimized for tested microorganism). Influent and effluent of each technical repeat were plated on MAC agar plates in triplicates to exclude non-lactose fermenters growth and false positive results, as well. Plates were enumerated based on PCM and results are presented in cfu/mL. The results are presented on Figure S2 and Table S1 in Extended Data Section.

## 1.6 Consumables’ Testing Procedure

The consumables testing experiment was designed to evaluate what kind of consumables should be used during process to avoid stickiness of bacteria to the consumable walls. Briefly, bacteria concentration in this experiment was set up as 1x10^2^ - 1x10^3^ bacteria per milliliter. Bacterial stock solution was set up 10^9^ bacteria/mL using OD meter and final concentration was obtained by serial 10x dilution of stock solution. Syringes, needles, syringes with needles, and tubing were filled up with bacteria and incubated 3 minutes in room temperature. Then, bacterial solution was released from tested consumables and wash with 3 mL of room temperature PBS diluted 1000x in UltraPure DI water w/o DNase and RNase. The whole volume was plated on agar plates (100 µL/plate), incubated overnight at 37 °C and enumerated next day to calculate cfu. Control sample (influent) bacteria incubated in the tube. All work was prepared in aseptic conditions. Obtained results are presented in Section 2.3.1 in the SI.

# 2. Extended Data:

## 2.1 Additional Validation of the Fluid Screen Method


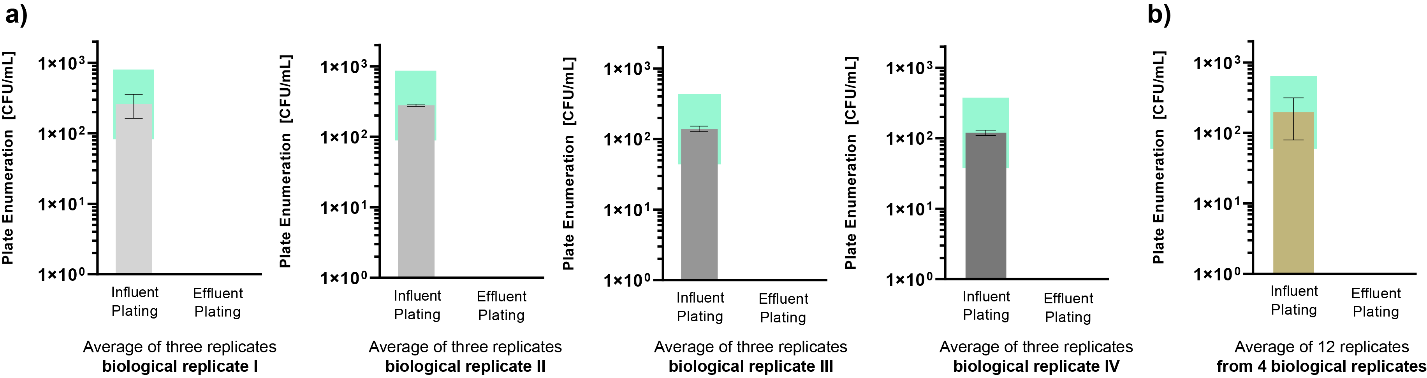


| **Plate-Count Method [cfu/mL]** | | | | | | | | | | | | |
| --- | --- | --- | --- | --- | --- | --- | --- | --- | --- | --- | --- | --- |
|  | Biological Replicate 1 | | | Biological Replicate 2 | | | Biological Replicate 3 | | | Biological Replicate 4 | | |
|  | Tech Rep 1  [Avg cfu/mL] | Tech Rep 2  [Avg cfu/mL] | Tech Rep 3  [Avg cfu/mL] | Tech Rep 1  [Avg cfu/mL] | Tech Rep 2  [Avg cfu/mL] | Tech Rep 3  [Avg cfu/mL] | Tech Rep 1  [Avg cfu/mL] | Tech Rep 2  [Avg cfu/mL] | Tech Rep 3  [Avg cfu/mL] | Tech Rep 1  [Avg cfu/mL] | Tech Rep 2  [Avg cfu/mL] | Tech Rep 3  [Avg cfu/mL] |
| **Neg_CTRL_** | 0 | 0 | 0 | 0 | 0 | 0 | 0 | 0 | 0 | 0 | 0 | 0 |
| **Influent**  **≤250 cfu/mL** | 330 | 240 | 200 | 280 | 420 | 130 | 130 | 290 | 0 | 190 | 150 | 20 |
| **Effluent** | 0 | 0 | 0 | 0 | 0 | 0 | 0 | 0 | 0 | 0 | 0 | 0 |
| **Cap_eff_ [%]** | **100** | **100** | **100** | **100** | **100** | **100** | **100** | **100** | **100** | **100** | **100** | **100** |

**Figure S2.** Results of capture efficiency experiments on the FS system. Plate Counting Method (PCM) quantification of bacteria in influent and effluent, after bacterial capture with the Fluid-Screen Chip. **(a)** Data is presented as a mean and +/-SD from three technical replicates per each biological replicate; **(b)** Data is presented as a mean and +/-SD from four biological replicates and their technical replicates, for a total of 12 tests. The turquoise bar represents the acceptable growth and viability variance range of +/- 0.5. log following the USP guidelines. In every experiment, the FS system captured 100% of bacteria, as evidenced by zero PCM growth in effluent samples. The 100% capture efficiency is maintained in a broad range of bacteria concentrations, including as high as 4.2x10^2^.

**Table S1.** The bacterial capture efficiency as tested by the Plate Counting Method (PCM) quantification. In every experiment, the FS system captured 100% of bacteria, as evidenced by zero PCM growth in effluent samples. The 100% capture efficiency is maintained in a broad range of bacteria concentrations, including as high as 4.2x10^2^.

In addition, to evaluate that Fluid-Screen chip can capture 100% of bacteria, *E. coli* stock sample was prepared in ~10^7^cfu/mL in PBS. The final concentration of Influent, up to 250 cfu/mL was achieved by serial tenfold dilutions of the stock sample. The 1 mL of sample was applied to the FS system under flow conditions and EF settings optimized for each tested microorganism. When the EF was still on, bacteria captured on FS system chip were stained with Sybr Green I for optical visualization and quantification (see Table 1 in the main text). Influent and Effluent of each technical replicate were plated on MAC agar plates to exclude non-lactose fermenters growth and false positive results. Plates were enumerated based on PCM and results are presented in cfu/mL. All nine replications (3 biological and 3 technical) on the FS system demonstrated reproducibility in 100% bacterial capture. The results of these experiments are summarized in Figure 3 and Table 1 in the main text. Additionally, sterility tests of the FS system, agar media, and aseptic sample preparation showed no growth of microorganisms at all.

## 2.2 Repeatability of bacterial quantification with the Fluid-Screen Method

Two biological samples with 12 technical repeats per each biological experiment on *E. coli*-8739 sample were performed to demonstrate the repeatability of the direct-on chip bacteria number quantification with the FS system. These results demonstrate the ability of the FS system to count the bacteria captured from a tested sample directly on the FS chip. The variance in results from the FS method measurement was caused by manual operation of the FS system. However, note that the FS bacterial counting error is low, and the FS method is more accurate than the required +/- 0.5 log recommended by the USP for a new method validation (Figure S3).


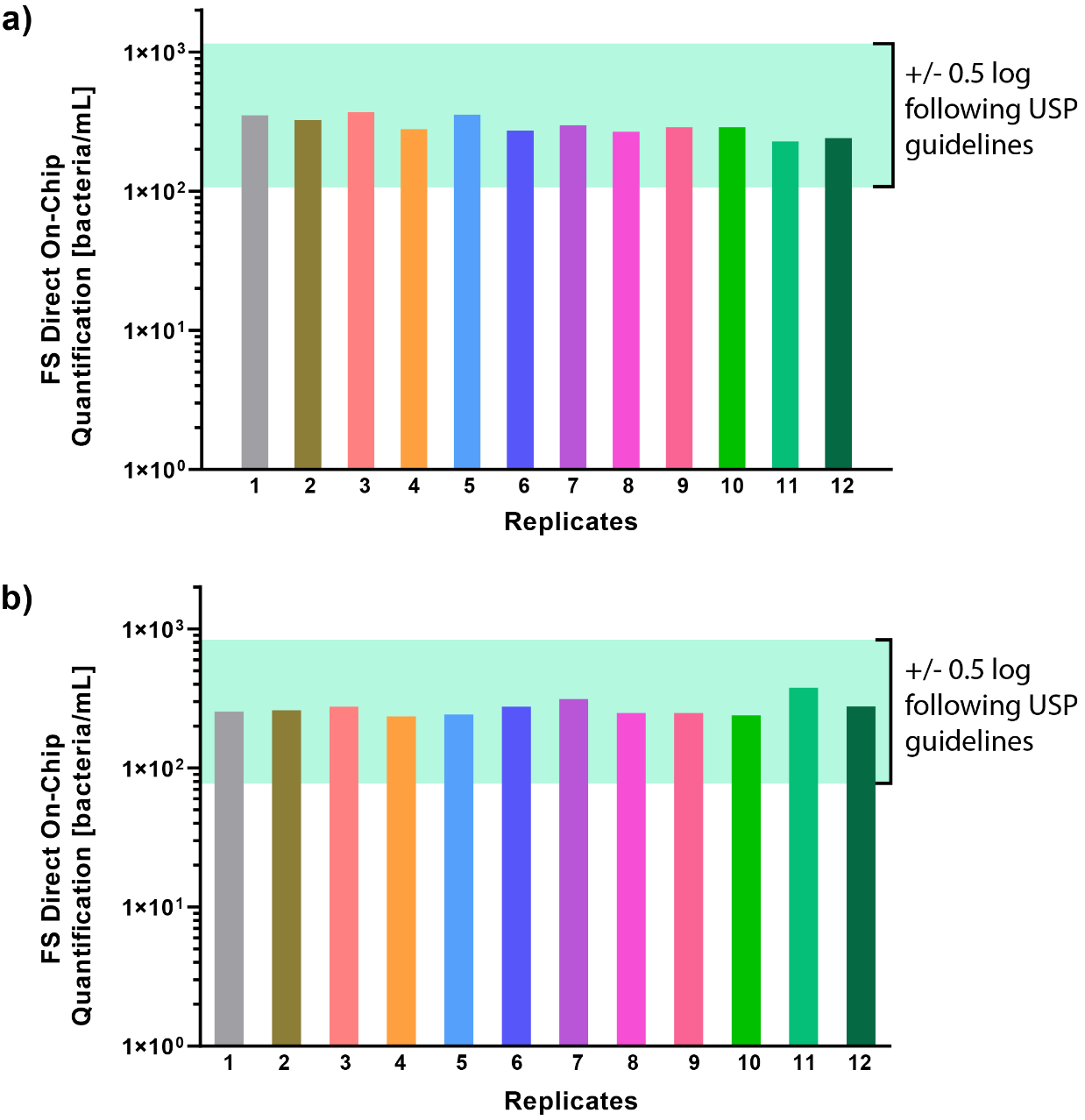


**Figure S3.** The repeatability of the bacterial capture and quantification on Fluid-Screen system. The Y axis: Total number of counted *E. coli*, X axis: repetitions of the Fluid-Screen capture experiment. The colored bars represent the number of bacteria captured in individual experiment. Fluid-Screen reliably captures bacterial cells, with a very high degree of repeatability. A sample of *E. coli*-8739 was analyzed 24 times in two sets of technical replicates, 12 technical each (a) and (b), demonstrating a high degree of system repeatability. The FS system results meet the +/- 0.5 log standards recommended by the USP for new methods. Note that +/- 0.5 log is calculated for the first grey bar repetition.

The repeatability in enumeration of the Fluid-Screen chips themselves is demonstrated on Figure S4. Eight different chips were used to directly quantify the number of captured *E. coli*-8739. There was no significant difference (ns) between biological replicates. Statistical analysis between the chips is summarized in Table S2. Again, the FS system demonstrated very high repeatability in the capture and quantification of bacteria.


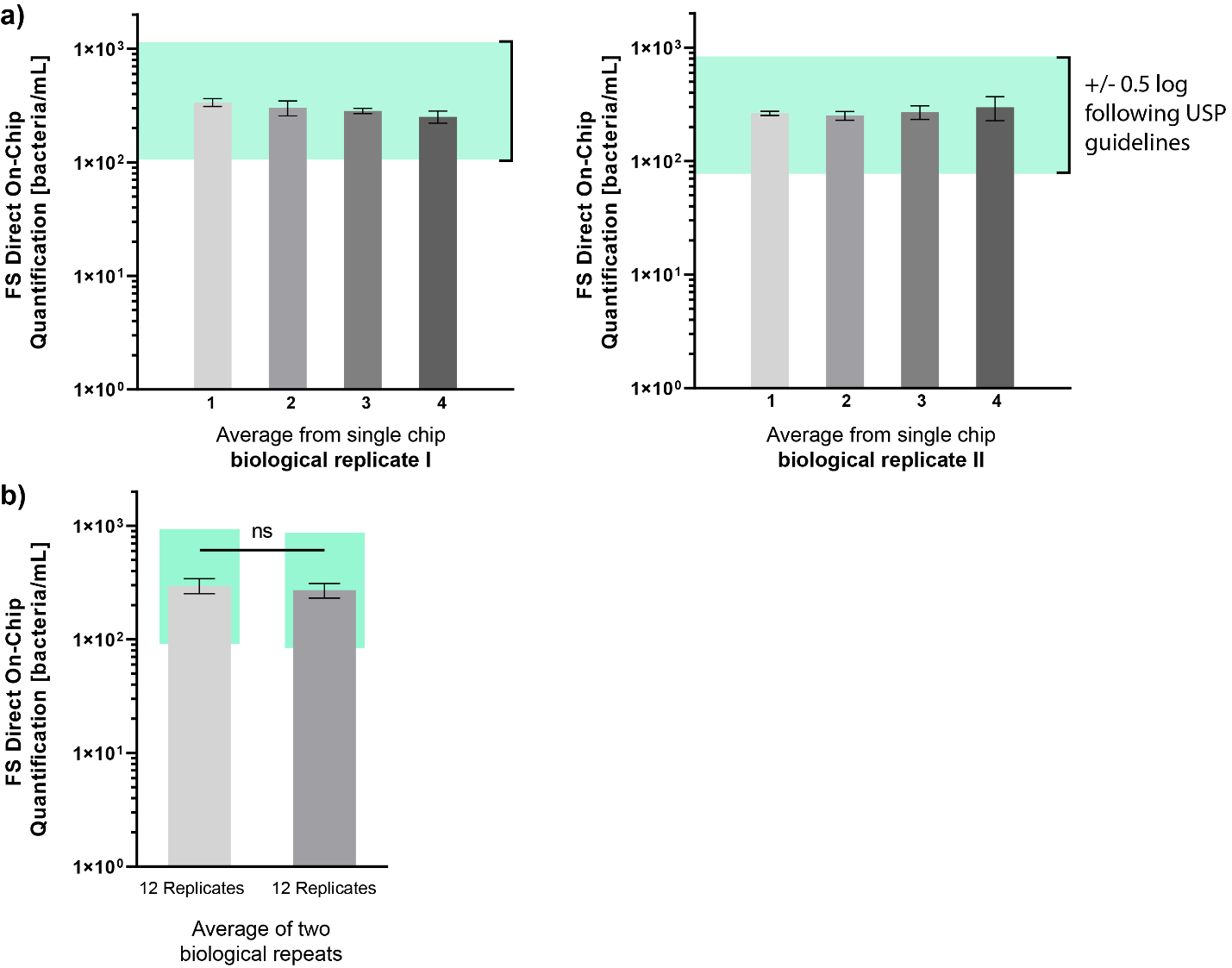


**Figure S4.** The directly FS-counted number of bacteria quantified with FS system on eight Fluid-Screen chips. **(a)** Each chip was used to count the bacteria three times (each chip has three channels), demonstrating a very high repeatability in chip capture and image processing performance. **(b)** Statistical comparison of two biological repeats, *p*=0.1438, unpaired t-test with Welch’s correction. The turquoise bar represents acceptable growth and viability variance range of +/- 0.5. log recommend by the USP.

| **Biological replicate 1** | | | | | |
| --- | --- | --- | --- | --- | --- |
|  |  | True number of bacteria | Mean | SD | %CV |
| Chip 1 | Channel 1 | 350 | 348.000 | 23.065 | 6.628 |
|  | Channel 2 | 324 |  |  |  |
|  | Channel 3 | 370 |  |  |  |
| Chip 2 | Channel 1 | 279 | 302.333 | 45.709 | 15.119 |
|  | Channel 2 | 355 |  |  |  |
|  | Channel 3 | 273 |  |  |  |
| Chip 3 | Channel 1 | 297 | 283.667 | 15.275 | 5.385 |
|  | Channel 2 | 267 |  |  |  |
|  | Channel 3 | 287 |  |  |  |
| Chip 4 | Channel 1 | 287 | 252.000 | 31.000 | 12.302 |
|  | Channel 2 | 228 |  |  |  |
|  | Channel 3 | 241 |  |  |  |
| **Biological replicate 2** | | | | | |
| Chip 1 | Channel 1 | 254 | 263.333 | 11.372 | 4.319 |
|  | Channel 2 | 260 |  |  |  |
|  | Channel 3 | 276 |  |  |  |
| Chip 2 | Channel 1 | 235 | 251.000 | 21.932 | 8.739 |
|  | Channel 2 | 242 |  |  |  |
|  | Channel 3 | 276 |  |  |  |
| Chip 3 | Channel 1 | 312 | 269.333 | 36.950 | 13.719 |
|  | Channel 2 | 248 |  |  |  |
|  | Channel 3 | 248 |  |  |  |
| Chip 4 | Channel 1 | 239 | 297.333 | 70.727 | 23.787 |
|  | Channel 2 | 376 |  |  |  |
|  | Channel 3 | 277 |  |  |  |

**Table S2.** The statistical analysis of the repeatability of the *E. coli* bacteria capture compared between several Fluid-Screen chips.

| **Example of microorganisms tested using Fluid-Screen technology** | | | | |
| --- | --- | --- | --- | --- |
| **Bacteria (Gram negative)** | **Type of Respiration** | **Media** | **Significance** | **References/Comments** |
| *A. laidlawii* | Aerobic | PBS  Mammalian cell culture medium | Common contaminant of growth media for cell culture | ATCC-23206-TTR  ^3^ |
| *B. caccae* | Anaerobic | PBS | Clinical pathogens | ^4^ |
| *B. fragilis* 25285 |  |  |  |  |
| *B. thetaiotamicron* 29148 |  |  |  |  |
| *B. vulgatus* 8482 |  |  |  |  |
| *E. coli DH5-α* | Aerobic | PBS  Fecal | Testing microorganisms | ATCC-67879 |
| *E. coli-GFP* S06 | Aerobic | PBS  Mammalian cell cultured medium | Testing microorganisms | ATCC-25922 |
| *E. coli* | Aerobic | PBS  Drug substance (concentrated protein solution) | Tests for microbial contamination | ATCC 8739  ^5^ |
| *E. coli* MC1060/pWTX594 | Aerobic | Water from the Charles River in Cambridge, MA | Testing microorganisms | ^6^ |
| *E. coli*-mCherry | Aerobic | PBS | Testing microorganisms | ATCC-MBA303  ^6^ |
| *L. pneumophila* 43109 | Anaerobic | DI Ultra-Pure Water | Water Contamination | ^7^ |
| *M. arginini* | Aerobic | PBS | Contamination during manufacturing process (cell therapy, tissue engineered products, cell culture) | ATCC-23838-TTR  ^8,9^ |
| *P. aeruginosa* | Aerobic | PBS  Drug substance (concentrated protein solution)  Mammalian cell culture medium | Tests for microbial contamination | ATCC-9027  ^5,8,10^ |
|  | | | | |
| **Bacteria (Gram positive)** | | | | |
| *A.* *kwangyangense* | Aerobic | PBS or Water from the Charles River in Cambridge, MA | Isolated from diesel contaminated costal sites | ATCC-700935  ^11^ |
| *B. cereus* 13061  (including endospores) | Facultative anaerobic | PBS | 1. Foodborne pathogen producing toxins, causing two types of gastrointestinal illness: the emetic syndrome and the diarrheal syndrome  2. Skin infections – keratitis  3. Non-sterile processing  4. Manufacturing environmental contamination e.g. dust | ^12–14^ |
| *B. circulans* 9500 | Aerobic | PBS | Pharmaceutical product contamination | ^15^ |
| *B. coagulans* BAA-738 | Aerobic | PBS | Health supplement (probiotic) | [www.fda.gov](http://www.fda.gov) |
| *B. megaterium* 14581 | Aerobic | PBS | Used as an alternative for high yield intra- and extracellular protein synthesis | ATCC-14581  ^16^ |
| *B. oleronius* 700005 | Aerobic | PBS | Establishment of Sterilization Conditions | Guidance on the Manufacture of Sterile Pharmaceutical |
| *B. subtilis* AG 147 | Aerobic | PBS  Fecal | Pharmaceutical product contamination | ATCC  ^8,10,17,18^ Guidance on the Manufacture of Sterile Pharmaceutical |
| *B. subtilis* 23857 | Aerobic | PBS | Establishment of Sterilization Condition |  |
| *B. subtilis* 6633 | Aerobic | PBS | Establishment of Sterilization Condition |  |
| *B. subtilis* 6051  (including endospores) | Aerobic | PBS | Pharmaceutical product contamination |  |
| *B. subtilis* CAL1388 | Aerobic | PBS | Testing microorganisms | ^6^ |
| *C. bolteae* BAA-613 | Anaerobic | PBS | Microbiome research | ATCC |
| *C. difficile* 43598 | Anaerobic | PBS | Causes life-threatening diarrhea | [www.cdc.gov](http://www.cdc.gov) |
| *C. perfringens* 13124 | Anaerobic | PBS | Pathogenic spore-forming bacteria, can be found on raw meat and poultry |  |
| *C. sporogenes* 3584 | Anaerobic | PBS | Testing organism  Sterility assurance  Testing  Testing disinfectants  Quality control of ENDO-SPOR™ hydrogen peroxide sterilization | 1. Applied Biosystems, MicroSeq Pharmaceutical Validation Panel.  2. Supplemental Efficacy: Sterilizers. Washington, DC: Environmental Protection Agency; EPA EPA DIS/TSS-9.  3. Sterilization of single-use medical devices incorporating materials of animal origin--Validation and routine control of sterilization by liquid chemical sterilants. Geneva (Switzerland): International Organization for Standardization/ANSI;ISO ISO 14160:1998.  4. Sterilization of single-use medical devices incorporating materials of animal origin --- Validation and routine control of sterilization by liquid chemical sterilants, Annex A. London, UK: British Standards Institution; British Standard BS EN ISO 14160:1998. |
| *E. faecalis* 19433 | Aerobic | PBS  Fecal | Testing organism  Media testing  Quality control strain  Reference material | ATCC |
| *E. faecalis* 47077 | Aerobic | PBS | Testing organism | ATCC |
| *P. acnes* 11827 B | Aerotolerant anaerobic | PBS | Human skin commensal, can be involved in the pathogenesis of acne | ^19^ |
| *S. aureus* | Aerobic | PBS  Drug substance (concentrated protein solution)  Mammalian cell culture medium | Tests for microbial contamination | ATCC-6538  ^5,10^ |
|  | | | | |
| **Fungus/Yeast/Molds** | | | | |
| *A. brasiliensis* | Aerobic | PBS  Drug substance (concentrated protein solution)  CHO cultured media | Tests for microbial contamination | ATCC-16404  ^8,10^ |
| *C. albicans* | Aerobic |  | Tests for microbial contamination | ATCC-10231  ^5,8,10^ |
| *S. cerevisiae* | Aerobic | PBS | Essential to winemaking  Foodborne spoilage microorganisms in commercial and fresh fruit juices | ^20,21^ |
|  | | | | |
| **Virus** | | | | |
| Human adenovirus 5 VR-5/Adenoid 75 | N/A | PBS  Mammalian cells cultured medium | Virucide testing  Respiratory research | ATCC-VR-5_70010153 |
|  | | | | |
| **Mammalian cells** | | | | |
| Chinese Hamster Ovary (CHO) | 5% CO_2_ | PBS  Mammalian cell cultured medium | Mammalian cells for biopharmaceutical production (e.g., antibody) | ^22,23^ |
|  |  |  |  |  |
| **Human cells** | | | | |
| Red blood cells | N/A | PBS | Medical testing |  |

**Table S3.** The diversity of selected microorganisms captured by the Fluid-Screen system.

## 2.3 Further Estimation of Losses of Bacterial Cells During the Bacterial Cell Capture and Separation with Fluid-Screen.

In this section we provide experimental evidence that bacteria are not lost during the FS capture and separation process and do not adhere to consumables used during FS operation. The design and the execution of the experiment is provided in Section 1.6 in the SI.

### 2.3.1 Results of the Consumables’ Testing

In this experiment we have showed that *E. coli*-GFP S06 bacteria used in the experiments do not form biofilm, i.e., do not adhere to any of used consumables like syringes, needles, tubes, tubing, and pipette tips during experiment, as illustrated in Figure S5.


**Fig. S5.** Estimation of bacterial losses on consumables during FS bacterial capture and separation procedure. Bacteria used in the test are not caught up (i.e., do not adhere) in the tube, pipette tips, syringe, syringe with needles and tubing even 40 inches long. The observed spread in numbers of bacterial colonies is from the plating techniques used during this experiment and not from the operation of FS. Because the volume of each tested bacterial solution sample was 3 mL and we cannot pour on agar plates more than 100 µL of liquid, each sample was plated on about 27 plates. Each point on the graph represents numbers of colonies from one agar plate. Spread is observed also because samples were distributed on agar plates using rolling techniques (standard plastic roller). Currently this part of FS work is being refined- we are now using a filtration method, which provides more consistent results as is shown below in Section 2.3.2 in the SI.

### 2.3.2 FS System Testing with Electric Field OFF

In the sets of FS experiments with electric field off, we have showed that bacteria used in the process do not form biofilm (i.e. do not adhere passively) on any of any part of the system. This experiment was specifically designed to evaluate that bacterium cannot be lost in the process. Samples containing a defined concentration of bacteria were prepared as described in FS Standard Protocol, Section 1.2.4. Influent and Effluent samples were treated according to protocol described in Section 1.2.5 - Filtration Method for Influent and Effluent Plating. Briefly, we have analyzed samples (Influent) with two different bacteria concentrations ~200 cfu/mL and ~20 cfu/mL. From experiments presented in section 2.3.1, we know that bacteria do not adhere to passively the tubes. Here, we test if the bacteria are not lost in the FS System while the electric field is set to off (Table S4). All experimental parameters are unchanged except that electric field is off. If the bacteria are not lost in the FS System (i.e., the bacteria do not adhere passively to FS system elements while the electric field is off), the number of bacteria in Influent should be equal with number of bacteria in Effluent. Obtained results for both bacteria concentrations are presented in the table below.

| **Sample** | **Concentration range** | **Influent** | **Effluent** |
| --- | --- | --- | --- |
|  |  | **Number of colonies**  **on TSA plates** | **Number of colonies on TSA plates** |
| 0.001xPBS | ~200 cfu/mL | 207 | 202 |
|  | ~20 cfu/mL | 26 | 19 |

**Table S4.** Comparison of the standard capture efficiency experiments (with electric field set to ON) to the experiments with electric field set to OFF, i.e. when bacteria were processed through the FS System electric field was off. The results presented indicate that bacteria are not lost during processing in the FS system.

Results presented on both Figure S5 and Table S4 show that neither consumables, nor the FS chip, nor whole FS system result in bacterial loss. Reduced number of colonies in the effluent of the sample with the lowest concentration (~20 cfu/mL) was observed because of the low concentration of spiked bacteria (below lowest range of colonies number accepted by USP for enumeration) (Table S4).

In summary, the control experiments for both the consumables and the FS system itself show that bacteria used in our experiments were not caught up in the tubes, tubing, pump valves, flow sensor, etc., and are not lost in the FS system during processing.

3. Supplementary References
1. Choi J-W, Rosset S, Niklaus M, Adleman JR, Shea H, Psaltis D. 3-dimensional electrode patterning within a microfluidic channel using metal ion implantation. *Lab Chip*. 2010;10(6):783-788.

2. McDonald JC, Duffy DC, Anderson JR, et al. Fabrication of microfluidic systems in poly (dimethylsiloxane). *Electrophor An Int J*. 2000;21(1):27-40.

3. Pharmacopeia US. 63—Mycoplasma Tests: A New Regulation for Mycoplasma Testing. USP 33/NF 28. In: *Usp Pharmacists’ Pharmacopeia; United States Pharmacopeial Convention: Rockville, MD, USA*. ; 2010:S3.

4. Wexler HM. Bacteroides: the good, the bad, and the nitty-gritty. *Clin Microbiol Rev*. 2007;20(4):593-621.

5. Pharmacopeia US. 62—Microbiological Examination of Nonsterile Products: Tests For Specified Microorganisms. USP 32. In: *Usp Pharmacists’ Pharmacopeia; United States Pharmacopeial Convention: Rockville, MD, USA*. ; 2009:S3.

6. Weber M, Markewich H, Mallick E, Schiltz M, Simon X. Broad-Range Bacterial Capture from Fluid-Samples: Implications for Amplification-Free Contamination Detection. *Sensors & Transducers*. 2016;203(8):40.

7. Oliva G, Sahr T, Buchrieser C. The life cycle of L. pneumophila: cellular differentiation is linked to virulence and metabolism. *Front Cell Infect Microbiol*. 2018;8:3.

8. Pharmacopeia US. 71—Sterility Tests / Microbiological Tests. USP 35. In: *Usp Pharmacists’ Pharmacopeia; United States Pharmacopeial Convention: Rockville, MD, USA*. ; 2009:S3.

9. Duguid J, Kielpinski G, du Moulin GC, Seymour B. Application of a Risk-Based Approach to Optimize a Rapid Mycoplasma Test for Cell Therapy and Tissue-Engineered Products. In: *AAPS Annual Meeting and Exposition*. Atlanta, GA; 2008.

10. Pharmacopeia US. 61—Microbiological examination of nonsterile products: Microbial enumeration tests. USP 31/FN26. In: *Usp Pharmacists’ Pharmacopeia; United States Pharmacopeial Convention: Rockville, MD, USA*. ; 2009:S3.

11. Kuczenski RS, Chang H-C, Revzin A. Dielectrophoretic microfluidic device for the continuous sorting of Escherichia coli from blood cells. *Biomicrofluidics*. 2011;5(3):32005.

12. Asaeda G, Caicedow G, Swanson C. Fried rice syndrome. *JEMS a J Emerg Med Serv*. 2005;30(12):30.

13. Pinna A, Sechi LA, Zanetti S, et al. Bacillus cereus keratitis associated with contact lens wear. *Ophthalmology*. 2001;108(10):1830-1834.

14. Payne DN. Microbial ecology of the production process. *Guid to Microbiol Control Pharm Med Devices*. 2006;482.

15. FDA. Kingston Pharma, LLC RECALLS " DG^TM^/Health NATURALS Baby Cough Syrup + Mucus” Because of Possible Health Risk. Center for Drug Evaluation and Research. https://tinyurl.com/yyupko3t. Published 2019.

16. Stammen S, Müller BK, Korneli C, et al. High-yield intra-and extracellular protein production using Bacillus megaterium. *Appl Environ Microbiol*. 2010;76(12):4037-4046.

17. PDA (Parenteral Drug Association). Technical Report No. 67: exclusion of objectionable microorganisms from nonsterile pharmaceuticals, medical devices, and cosmetics. 2014.

18. Cundell T. Exclusion of Objectionable Microorganisms from Non‐sterile Pharmaceutical Drug Products. *Pharm Microbiol Qual Assur Control Pract Guid Non‐Sterile Manuf*. 2019:371-400.

19. McLaughlin J, Watterson S, Layton AM, Bjourson AJ, Barnard E, McDowell A. Propionibacterium acnes and acne vulgaris: new insights from the integration of population genetic, multi-omic, biochemical and host-microbe studies. *Microorganisms*. 2019;7(5):128.

20. Mendoza LM, Fernandez de Ullivarri M, Raya RR. Saccharomyces cerevisiae: A key yeast for the wine-making process. 2018.

21. Brice C, Cubillos FA, Dequin S, Camarasa C, Martinez C. Adaptability of the Saccharomyces cerevisiae yeasts to wine fermentation conditions relies on their strong ability to consume nitrogen. *PLoS One*. 2018;13(2):e0192383.

22. Shin SW, Lee JS. CHO Cell Line Development and Engineering via Site-specific Integration: Challenges and Opportunities. *Biotechnol Bioprocess Eng*. 2020:1-13.

23. Scarcelli JJ, Shang TQ, Iskra T, Allen MJ, Zhang L. Strategic deployment of C HO expression platforms to deliver Pfizer’s Monoclonal Antibody Portfolio. *Biotechnol Prog*. 2017;33(6):1463-1467.
